# An evaluation of thermal tolerance in six tardigrade species in an active and dry state

**DOI:** 10.1242/bio.060485

**Published:** 2024-09-25

**Authors:** Jacob Loeffelholz, Emma Meese, Ilaria Giovannini, Karsyn Ullibarri, Sogol Momeni, Nicholas Merfeld, Jessica Wessel, Roberto Guidetti, Lorena Rebecchi, Thomas C. Boothby

**Affiliations:** ^1^ University of Wyoming, Department of Molecular Biology, Laramie, WY 82071, USA; ^2^ University of Modena and Reggio Emilia, Department of Life Sciences, Modena 41125, Italy; ^3^ University of Alabama, Department of Biological Sciences, Tuscaloosa, AL 35487, USA; ^4^ University of Iowa, Tippie College of Business, Iowa City, IA 52242, USA; ^5^ Independent researcher, Dubuque, Iowa 52001, USA

**Keywords:** Anhydrobiosis, Desiccation tolerance, Heat shock, Tardigrade, Thermal tolerance

## Abstract

Tardigrades are known for their ability to survive extreme conditions. Reports indicate that tardigrade thermal tolerance is enhanced in the desiccated state; however, these reports have almost always used a single tardigrade species and drying/heating methods vary between studies. Using six different species of tardigrades we confirm that desiccation enhances thermal tolerance in tardigrades. Furthermore, we show that differences in thermal tolerance exist between tardigrade species both when hydrated and desiccated. While *Viridiscus viridianus* survives the highest temperatures in the hydrated state of any species tested here, under hydrated conditions, the thermal tolerance of *V. viridianus* is restricted to an acute transient stress. Furthermore, unlike other stresses, such as desiccation, where mild initial exposure preconditions some species to survive subsequent harsher treatment, for *V. viridianus* exposure to mild thermal stress in the hydrated state does not confer protection to harsher heating. Our results suggest that while tardigrades have the capacity to tolerate mild thermal stress while hydrated, survival of high temperatures in a desiccated state is a by-product of tardigrades' ability to survive desiccation.

## Teaser

**Summary:** Using six species of tardigrade we observe that thermal tolerance varies by species, and consistent with previous work we find that anhydrobiosis increases thermal tolerance relative to hydrated/active conditions.

## INTRODUCTION

Tardigrades are microscopic, invertebrate animals in the phylum Tardigrada. Currently, about 1500 species of tardigrades are known to be extant (Degma and Guidetti, 2024). Commonly referred to as “water bears”, tardigrades are considered some of nature's toughest animals and have received much scientific and public interest for their ability to tolerate harsh environmental conditions including near complete desiccation, intense radiation, extremes in temperature, low oxygen conditions, and even the vacuum of outer space (Hagelbäck and Jönsson, 2023; Horikawa et al., 2006, 2013; Jönsson et al., 2008; Kinchin, 1994; Rebecchi et al., 2009a; Rizzo et al., 2015).

To survive several of these stresses, tardigrades utilize a strategy known as cryptobiosis (‘hidden life’), which can further be classified by the type of stress that an organism is experiencing. These states include cryobiosis, chemobiosis, anoxybiosis, osmobiosis, and anhydrobiosis (Møbjerg et al., 2011; Nelson et al., 2010).

One relatively understudied tolerance that tardigrades are reported to have is that of thermal or heat tolerance. Thermal tolerance in tardigrades has been investigated in several studies which generally indicate that tardigrades in a desiccated state are more thermal tolerant than those in an active, hydrated state (Giovannini et al., 2023; Halberg et al., 2013; Hengherr et al., 2009; Horikawa et al., 2012; Li and Wang, 2005a,b; Neves et al., 2020a,b; Rebecchi et al., 2009b; Vecchi et al., 2021). In these studies, survival is typically scored as observation of coordinated movement and/or response to stimuli. Interestingly in several studies examining thermal tolerance in active tardigrades, survival was seen to increase with prolonged recovery time (Rebecchi et al., 2009b). While tardigrades can be acclimatized or ‘preconditioned’ to survive some stresses, pre-exposure of tardigrades to higher temperatures has been observed to acclimatize tardigrades modestly at best (Neves et al., 2020a), but in some cases pretreatment with mild heating was observed to be detrimental to thermal tolerance (Li and Wang, 2005a). While these pioneering works have begun to shed light on the phenomenon of thermal tolerance in tardigrades, many of them have focused on a single tardigrade species and/or use varying methods of heating, drying, and scoring for survival.

In the current study, we examine the effects of thermal stress on the survival of six different species of tardigrades in both the active and desiccated state that span both classes of tardigrades (Eutardigrada and Heterotardigrada), and which were originally collected from locations with diverse humidities and average temperatures. We find that thermal tolerance varies significantly between species, regardless of hydration state. We confirm that in five of the six species tested, dry animals are able to tolerate significantly higher temperatures than their hydrated counterparts. Furthermore, we find that response to stimuli and coordinated movement increased in several species when examined 72 h post-heating relative to the same specimens examined 1 h post-heating. We identify *Viridiscus viridianus*, as the species in our study with the greatest thermal tolerance in the hydrated state. We find that this species can only survive a relatively acute thermal stress, and that heating at a lower temperature does not prime *V. viridianus* specimens to survive more severe thermal stress. Furthermore, we see that repeated heating does not affect survival of this species. From these experiments we suggest that robust tardigrade thermal stress tolerance displayed by some species in the dry state is a by-product of their ability to survive desiccation rather than an intrinsic ability to tolerate high temperatures. Furthermore, as has been shown by previous studies, tardigrades' ability to survive heating in the hydrated state is mild at best and only allows for exposure to elevated temperatures for short periods of time (Giovannini et al., 2023; Halberg et al., 2013; Hengherr et al., 2009; Horikawa et al., 2012; Li and Wang, 2005a,b; Neves et al., 2020a,b; Rebecchi et al., 2009b; Vecchi et al., 2021). These results provide insights into the evolution of thermal tolerance in some desiccation tolerant tardigrades via cross-tolerance as well as supporting the assertion that despite their ability to survive several environmental extremes, tardigrades may in fact be a good indicator species for global temperature increases associated with climate change.

## RESULTS

### Tardigrade species display differential thermal tolerance in an active state

**Fig. 1.**
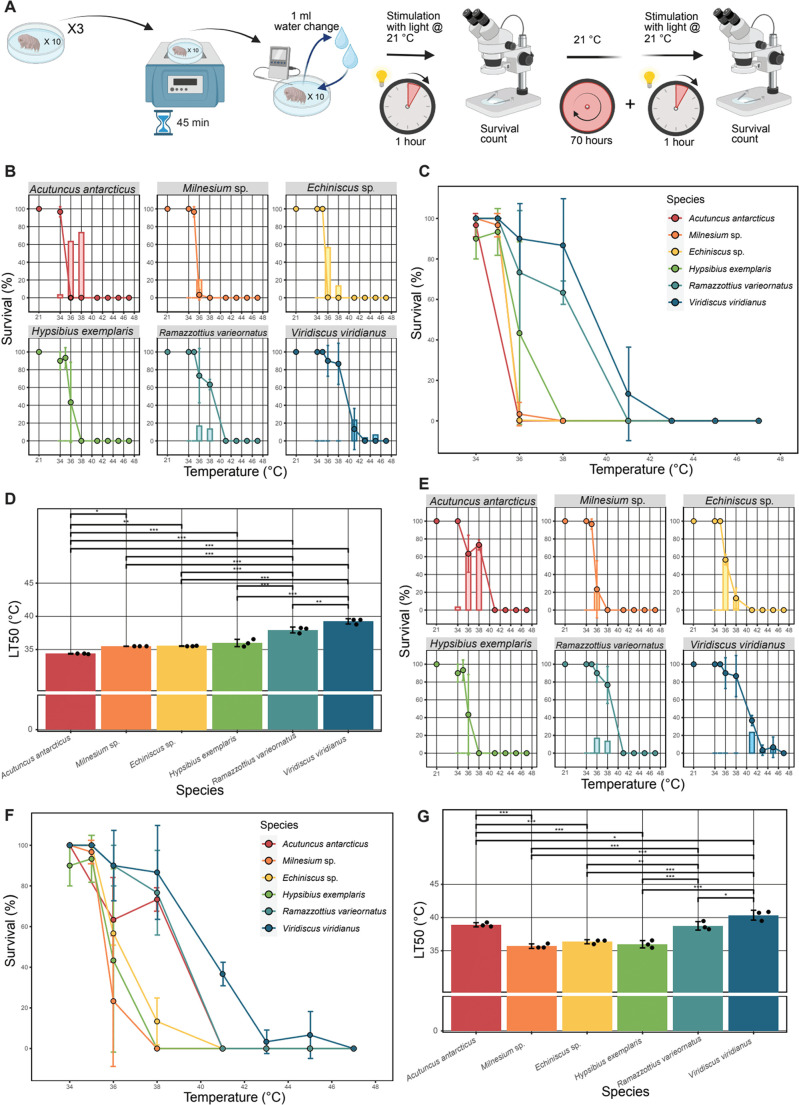
**Hydrated tardigrades differ in their thermal tolerance.** (A) Diagram of experimental setup for heating of active tardigrades. (B) Percent activity of hydrated specimens observed at 1 h post stress. Columns represent the difference in apparent survival at 72 h and 1 h observation, calculated by subtracting observed activity at 72 h from observed activity at 1 h (72 h activity - 1 h activity). Error bars on points show standard deviation. (C) Overlay of percent survival of all species as observed at 1 h post stress. (D) LT50 for hydrated species calculated from 1 h post stress survival data. (E) Percent survival of hydrated specimens observed at 72 h post stress. Columns represent the difference in apparent survival at 72 h and 1 h observation calculated by subtracting observed activity at 72 h from observed 1 h activity (72 h activity - 1 h activity). Error bars on points show standard deviation. (F) Overlay of percent survival of all species as observed at 72 h post stress. (G) LT50 for hydrated species calculated from 72 h post stress survival data. All data gathered from three replicates of *n*=10 tardigrades. For statistical significance one-way ANOVA with Tukey’s post-hoc tests were used, *=*P*-value >0.05, **>0.01, ***>0.005.

To assess the thermal tolerance of active tardigrades, we made use of three cultured and three environmentally sampled species of tardigrade. Cultured species included *Hypsibius exemplaris*, *Mazzites varieornatus*, and *Acutuncus antarcticus*. Field collected samples included *V. viridianus*, *Echiniscus* sp., and *Milnesium* sp. (see methods for genus level identification). Hydrated specimens of each species were heated from room temperature (∼21°C) to increasingly high final temperatures (from a minimum of 34°C to a maximum of 47°C) over the course of 45 min, after which their water was exchanged, and specimens were left at room temperature (∼21°C). These temperatures (Neves et al., 2020a; Rebecchi et al., 2009b) and incubation time (Loeffelholz et al., 2021) were chosen based on previous studies of tardigrade thermal tolerance in the hydrated state. After 1 h and 72 h specimens were examined for activity, which was gauged by assessing response to stimuli (light) and coordinated movement (Fig. 1A). 1 h was selected as an observation time as it allows for immediate assessment of tardigrade activity following heating. 72 h was selected as it allows for assessment of longer-term survival following heating. Control specimens from each species tested showed 100% activity when kept under ambient conditions (21°C) for 1 h (Fig. 1A). After 1 h, different tardigrade species displayed dramatically different responses to heating (Fig. 1B,C). *A. antarcticus* and *Echiniscus* sp. maintained activity only at temperatures below 36°C. *Milnesium* sp. and *H. exemplaris* showed activity after heating to 36°C. *R. varieornatus* were active at temperatures up to 38°C and *V. viridianus* performed best tolerating temperatures to 41°C. Differences in survival were also assessed in terms of the lethal temperature 50 (LT50), the temperature at which 50% of the specimens are expected to perish. When comparing these temperatures, we find that there were significant differences in the LT50 between many of the tested species (Fig. 1D; Table 1). After survival assessment at 1 h, tardigrades were given an additional 71 h of recovery and then assessed again. Again, all control animals kept at 21°C showed activity at this time point (Fig. 1E). While trends in survival largely held consistent when examining specimens 72 h post-heating, we found that with prolonged recovery some animals which appeared to be dead after 1 h post-heating were now active (Fig. 1E-G). This finding is consistent with previous reports that response to stimuli and coordinated movement after longer-term assessment improve in several cases relative to 1 h samples (Rebecchi et al., 2009b). Interestingly, similar trends have been reported before in tardigrades, for example in recovery from irradiation (Jönsson et al., 2016), freezing (Guidetti et al., 2011a) and drying (Nagwani et al., 2024). In all species examined, inactive 1 h specimens, regardless of whether they recovered or did not recover at 72 h, displayed the same morphological characteristics of being turgid with their appendages outstretched and were visually indistinguishable from one another.

**Table 1.**
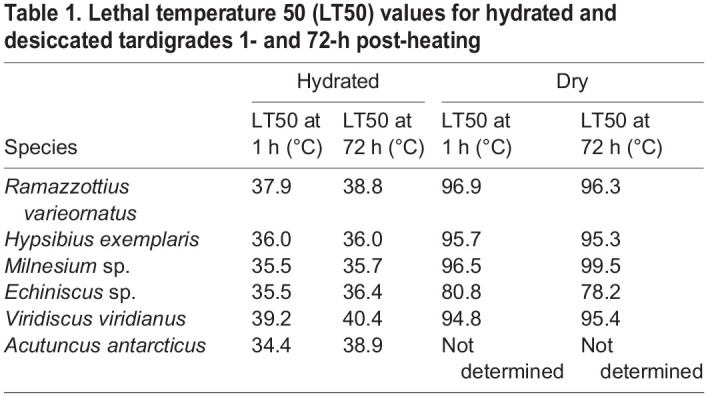
Lethal temperature 50 (LT50) values for hydrated and desiccated tardigrades 1- and 72-h post-heating

These results demonstrate that thermal tolerance in active hydrated tardigrades varies between species. Furthermore, our results confirm that tardigrades suspend some normal life processes upon sufficient heating (e.g. coordinated movement and response to photostimulation) and do not always immediately resume these processes upon cessation of thermal stress.

### *V. viridianus* survives repeated short-term thermal stress

**Fig. 2.**
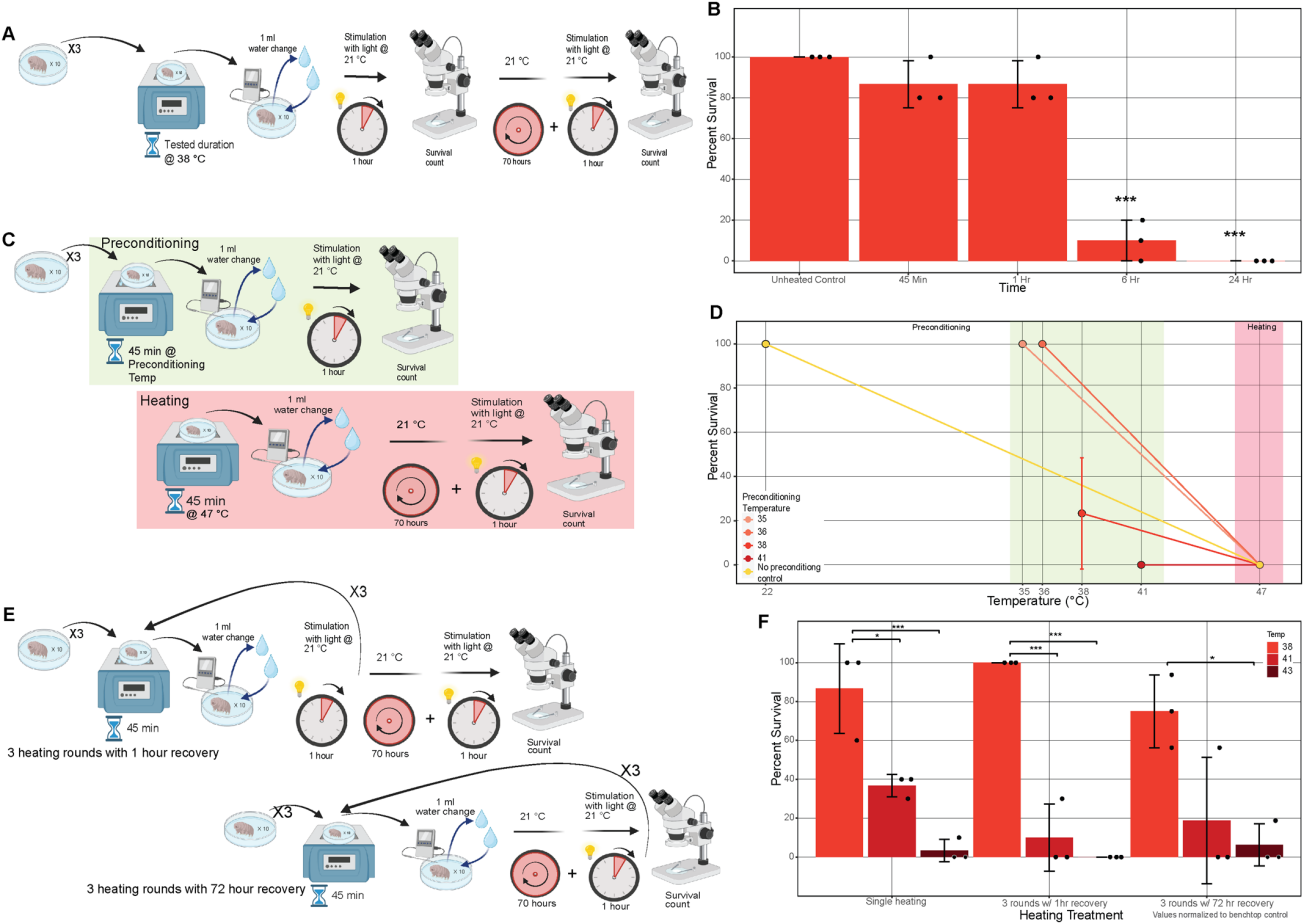
**The thermal tolerance of *V. viridianus* is short-term and does not improve with pre-exposure.** (A) Diagram of experimental setup for heating active tardigrades for extended time periods. (B) Percent survival of *V. viridianus* during heating for extended periods of time at 38°C. Statistical comparisons shown are relative to non-heated control samples (C) Diagram of experimental setup for preconditioning of active tardigrades. (D) Survival of *V. viridianus* during low temperature preconditioning (green) and subsequent heating at 47°C (red). No differences exist between preconditioned tardigrades and non-preconditioned control. (E) Diagram of experimental setup for repeated heating experiments. (F) Differences in percent survival of *V. viridianus* over multiple heating periods. Data for 72-h recovery samples are shown normalized to a 9-day benchtop control to account for natural death of specimens kept in water without feeding. Non-significant differences not shown. For statistical significance one-way ANOVA with Tukey’s post-hoc tests were used, *=*P*-value >0.05, **>0.01, ***>0.005.

To investigate whether the thermal tolerance of tardigrades in an active state also persists over extended periods, we exposed *V. viridianus*, which survived the highest temperatures while active, to 38°C temperatures over several different time intervals (Fig. 2A). Consistent with our original experiments, *V. viridianus* robustly survived heating to 38°C for 45 min and showed the same survival at 1 h. However, following 6 h of heating there was a precipitous drop in survival and after 24 h of heating no specimens survived (Fig. 2B), suggesting that tardigrade thermal tolerance is only to acute and not prolonged heating.

In addition to surviving several stresses, such as drying, in some cases exposure to a mild stress can condition tardigrades to survive a more severe stress that they otherwise would not tolerate. An example is desiccation, where slow drying of some specimens preconditions them to survive a faster and harsher drying regime that they normally would not tolerate (Boothby et al., 2017; Kondo et al., 2015; Wright, 1989). To examine whether a mild thermal stress can precondition tardigrades to survive a more severe one, we exposed *V. viridianus* specimens to several lower temperatures (35-41°C) under which they survive to varying degrees in the active state. Following this initial 45-min heating period animals were given 1 h of recovery time and we then assessed their ability to survive exposure to a non-permissive temperature (47°C) (Fig. 2C). One hour of recovery time was chosen due to the nature of preconditioning for other stresses. When tardigrades are preconditioned for drying, they upregulate mediators of desiccation and the presence of those mediators is what allows them to survive harsher drying conditions (Kondo et al., 2015). With this concept in mind, we utilized only 1 h to make sure that any upregulated mediators of thermal tolerance would still have the likelihood of being present when heated at a higher temperature. We observed that for lower preconditioning temperatures (35-36°C), all animals survived preconditioning, but none survived higher temperature heating. For higher temperature preconditioning (38-41°C), we observed that not all specimens survived pretreatments and that those that did survive pretreatment did not survive subsequent exposure to 47°C. As expected, control tardigrades that experienced no pre-heating also did not survive exposure to 47°C (Fig. 2D). This data demonstrates that heating at lower permissive temperatures does not prime *V. viridianus* to tolerate exposure to more severe thermal stress.

**Fig. 3.**
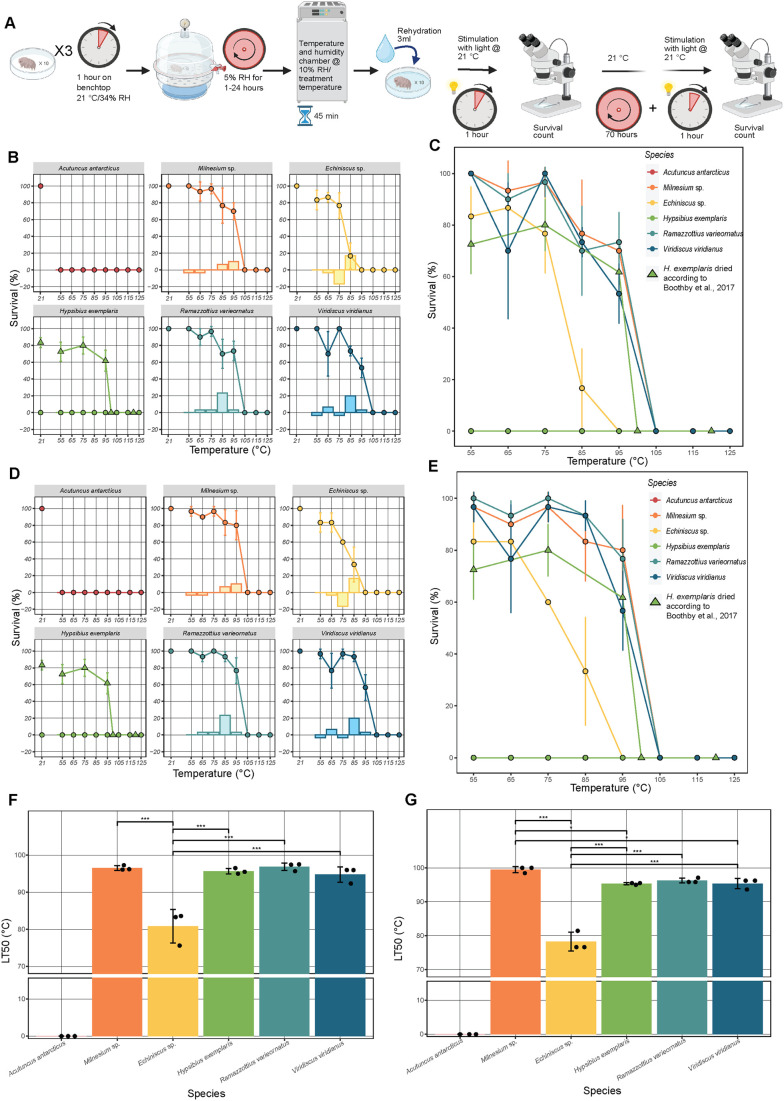
**Thermal tolerance varies among tardigrade species in the dry state.** (A) Diagram of experimental setup for heating of desiccated tardigrades. (B) Percent activity of desiccated specimens observed at 1 h post stress. Columns represent the difference in apparent survival at 72 h and 1 h calculated by subtracting observed activity at 72 h from observed 1 h activity (72 h activity - 1 h activity). Error bars on points show standard deviation. (C) Overlay of percent survival of all species as observed at 1 h post stress. (D) Percent survival of desiccated specimens observed at 72 h post stress. Columns represent the difference in apparent survival at 72 h and 1 h calculated by subtracting observed activity at 72 h from observed 1 h activity (72 h activity - 1 h activity). Error bars on points show standard deviation. (E) Overlay of percent survival of all species as observed at 72 h post stress. (F) LT50 for desiccated species calculated from 1 h post stress survival data. (G) LT50 for desiccated species calculated from 72 h post stress survival data. LT50 was not determined for species that did not survive any tested heating. *H. exemplaris* dry LT50 calculated from data collected in Boothby et al. (2017); fig. 7D (triangles). All data gathered from three replicates of *n*=10 tardigrades. For statistical significance one-way ANOVA with Tukey’s post-hoc tests were used, *=*P*-value >0.05, **>0.01, ***>0.005.

Finally, we wondered whether heat stress at permissive temperatures could improve tolerance to repeated heat at that temperature. To probe this, we again made use of *V. viridianus*. First, we heated specimens to 38, 41 or 43°C three times for 45 min with 1 h of recovery at room temperature (21°C) in between. At all temperatures tested, heating *V. viridianus* three times back-to-back (Fig. 2E) did not negatively affect survival significantly compared to heating *V. viridianus* once at the same temperature (Fig. 2F). A similar result was obtained when *V. viridianus* was heated three times with a 72-h recovery period between each heat stress (Fig. 2F). Allowing the tardigrades 72 h of recovery per heating treatment meant that they were without algae for 9 days, thus the survival of these samples was normalized to survival of non-heated tardigrades left at room temperature without feeding over the same time period. These results are of interest since each temperature tested here results in some loss of viability after only a single stress. One might expect that without preconditioning survival would decrease by the same amount (or more) with each additional instance of the stress. However, these expected decreases in survival were not observed after the first heating event, for specimens heated three times with either 1 h or 72 h of recovery, suggesting that that first heating event preconditioned the animals to survive subsequent heating events at that temperature. If preconditioning had not occurred, we would have expected a continual decline in survival with each subsequent heating event, but we did not observe this (Fig. 3F).

Combined these results demonstrate that *V. viridianus* only tolerates thermal stress for a short duration while in the hydrated state. Furthermore, thermal stress does not appear to precondition the animals to survive heating at non-permissive temperatures while active (at least under the conditions tested) but does seem to precondition animals to survive repeated stress at permissive temperatures.

### Desiccation increases thermal tolerance in most tardigrade species

While most active tardigrades tested here survived only mild thermal stress in the hydrated state, previous studies have reported a dramatic increase in thermal tolerance of tardigrades in the desiccated state (Neves et al., 2020a). To examine how desiccation influences the thermal tolerance of tardigrades from different classes (Heterotardigrada versus Eutardigrada) and from different geographical sites of origin, we dried each of our six species prior to subjecting them to heating at various temperatures (Fig. 3A). It should be noted that for consistency, all tardigrade species were dried using the same desiccation regime (see Materials and Methods). This desiccation regime allows for robust survival of our controls, specimens dried and stored at room temperature, with the exception of *H. exemplaris* (Fig. 3B-E), as *H. exemplaris* requires preconditioning to survive drying (Boothby et al., 2017; Kondo et al., 2015; Wright, 1989). In the case of *H. exemplaris*, thermal survival data from figure 7D in Boothby et al. (2017), which was generated using a preconditioning drying regime, is shown here for comparative purposes. Readers should note that Boothby et al. (2017) refers to *H. exemplaris* as *Hypsibius dujardini* as was the convention at the time.

**Fig. 4.**
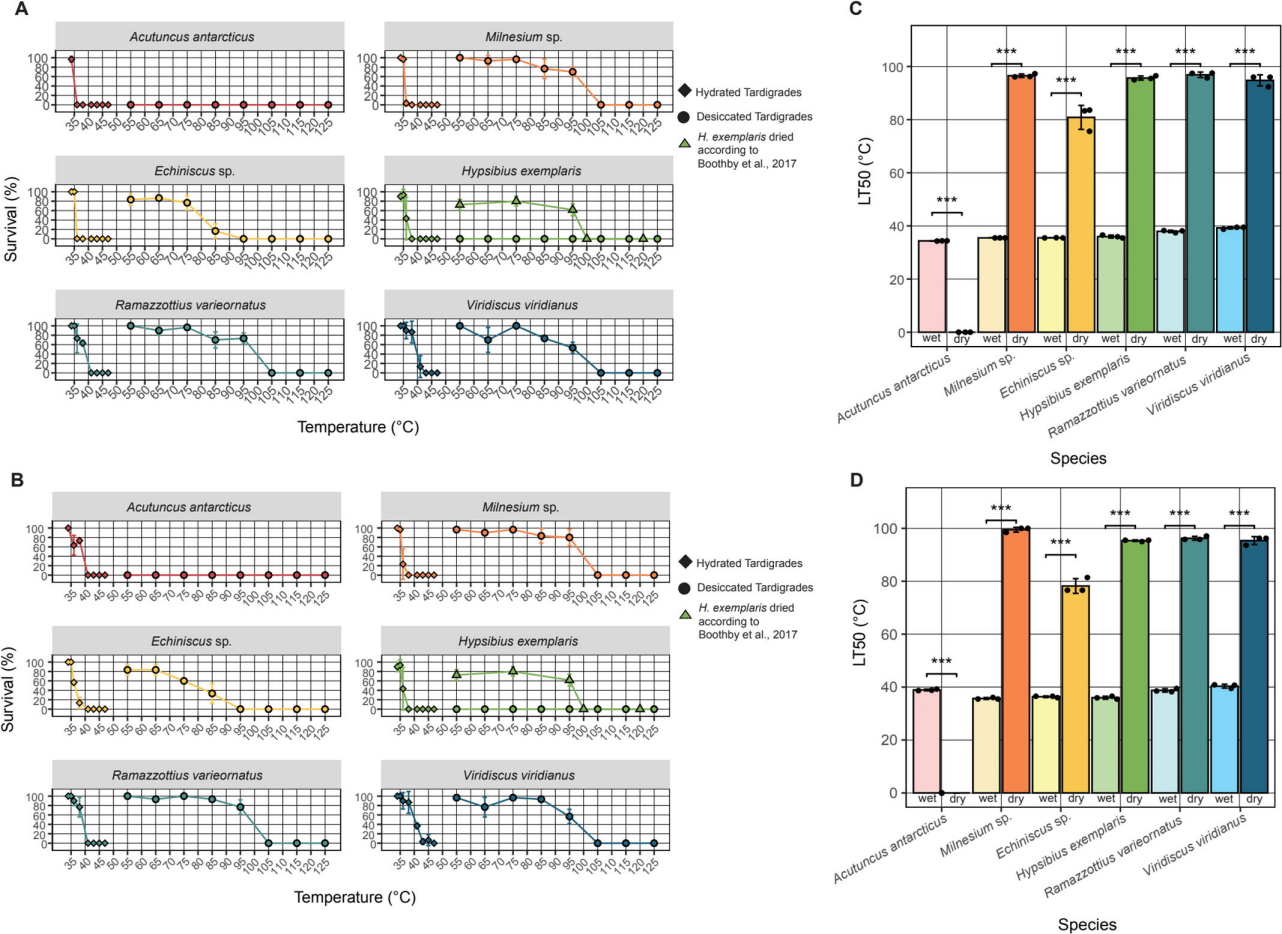
**Thermal tolerance of tardigrades differs dramatically between the hydrated and desiccated state.** (A) Percent activity/survival of hydrated species (diamonds) and desiccated (circles) species observed at 1 h post stress (for hydrated specimens) and post rehydration (for desiccated specimens). *H. exemplaris* data from Boothby et al. (2017); fig. 7D (triangles) presented for comparison. (B) Percent survival of hydrated (diamonds) and desiccated (circles) species observed at 72 h post stress (for hydrated specimens) and post rehydration (for desiccated specimens). *H. exemplaris* data from Boothby et al. (2017); fig. 7D (triangles) presented for comparison. (C) Comparison of hydrated and desiccated LT50 temperatures from 1 h post stress/rehydration survival. (D) Comparison of hydrated and desiccated LT50 temperatures from 72 h post stress/rehydration survival. *H. exemplaris* dry LT50 calculated from data collected in Boothby et al. (2017) (fig. 7D). All data gathered from three replicates of *n*=10 tardigrades. For statistical significance one-way ANOVA with Tukey’s post-hoc tests were used, *=*P*-value >0.05, **>0.01, ***>0.005.

As with active tardigrades, desiccated species were exposed to elevated temperatures for 45 min and their activity was recorded after 1 h (Fig. 3B,C) and then again at 72 h post-heating (Fig. 3D,E). Additionally, the LT50 for both 1 and 72 h post-heating was calculated and compared (Fig. 3F,G, Table 1). Robust survival was observed in five of six species tested. Interestingly, *A. antarcticus* did not survive heating at any tested temperature while desiccated. *Echiniscus* sp. survived up to 85°C, while *Milnesium* sp., *R. varieornatus*, preconditioned *H. exemplaris*, and *V. viridianus* survived up to 95°C. As with tardigrades subjected to heating in the active state, some species display greater response to stimuli and coordinated movement at 72 h relative to 1 h post-heating (Fig. 3B,D). Unlike with hydrated samples however, some species did show decreased response to stimuli and coordinated movement at 72 h versus 1 h post-heating (Fig. 3B,D). As expected, the overall survival of desiccated samples is generally higher than hydrated samples both after 1 h (Fig. 4A) and 72 h (Fig. 4B) of recovery. Similarly, at both 1 h (Fig. 4C) and 72 h (Fig. 4D) of recovery dry tardigrades showed significantly higher LT50 values compared to their hydrated counterparts (Table 1). These results suggest that thermal tolerance in many species of tardigrades is enhanced by entry into the dry state.

### Thermal tolerance in tardigrades is not correlated with collection site macroscale environmental conditions

**Fig. 5.**
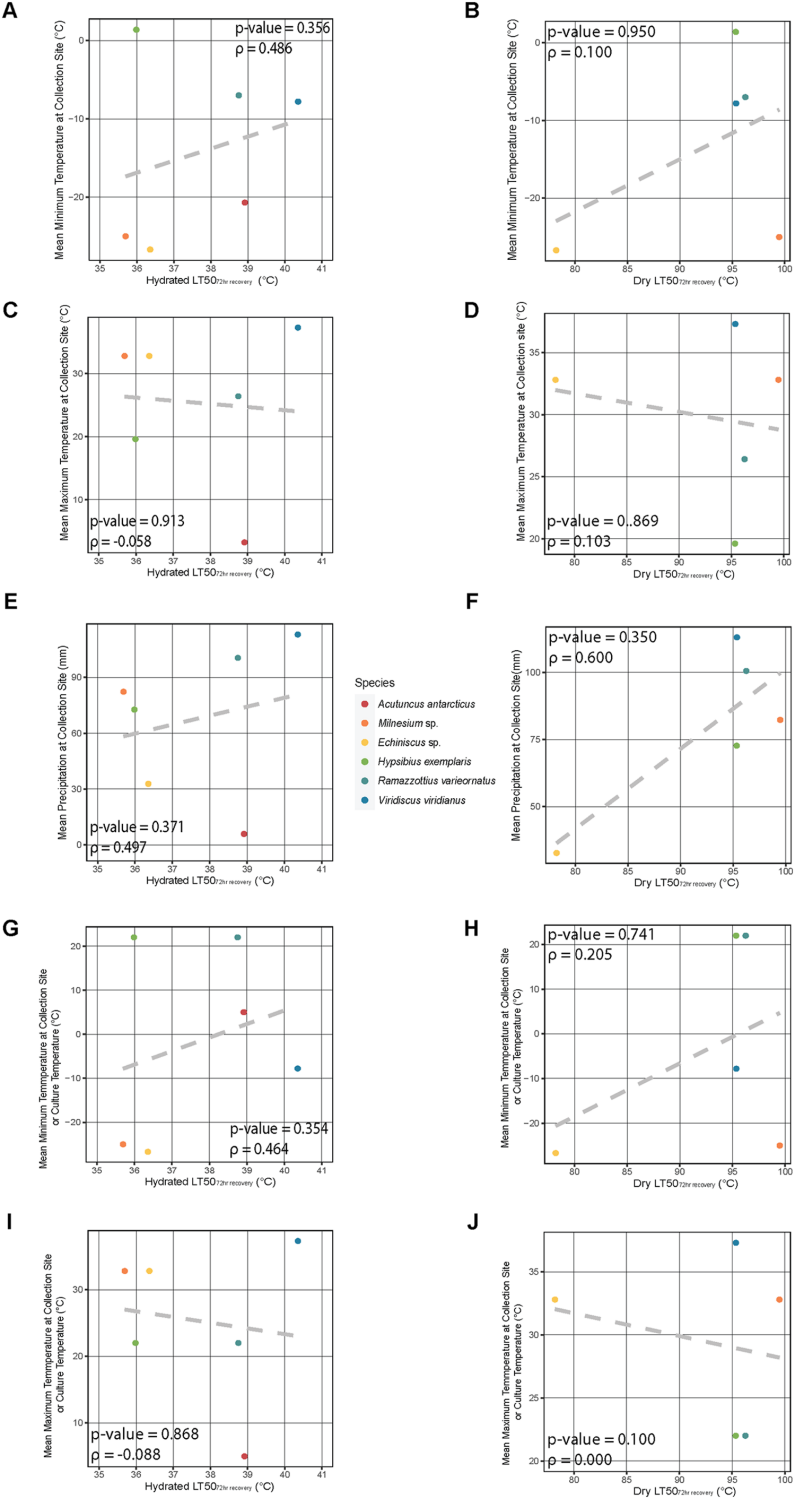
**Thermal tolerance in tardigrades does not correlate significantly with the temperature and precipitation data from their sites of collection.** (A) Correlation of LT50, from 72 h survival data, of wet (hydrated) specimens to mean minimum temperature of their collection site. (B) Correlation of LT50, from 72 h survival data, of dry specimens to mean minimum temperature of their collection site. (C) Correlation of LT50, from 72 h survival data, of wet specimens to mean maximum temperature of their collection site. (D) Correlation of LT50, from 72 h survival data, of dry specimens to mean maximum temperature of their collection site. (E) Correlation of wet specimens to mean monthly rainfall of their collection site. (F) Correlation of LT50, from 72 h survival data, of dry specimens to mean monthly rainfall of their collection site. (G) Correlation of LT50 of wet specimens to the mean minimum temperature at the collection site (for field samples) or culture temperature (for samples from culture). (H) Correlation of LT50 of dry specimens to the mean minimum temperature at the collection site (for field samples) or culture temperature (for samples from culture). (I) Correlation of LT50 of wet specimens to the mean maximum temperature at the collection site (for field samples) or culture temperature (for samples from culture). (J) Correlation of LT50 of dry specimens to the mean maximum temperature at the collection site (for field samples) or culture temperature (for samples from culture). LT50 of dry specimens of *H. exemplaris* calculated from data presented in Fig. 7D of Boothby et al. (2017). Spearman's ranked correlation coefficient (ρ) and *P*-value are displayed on graphs.

Due to the differences in survival across species, we wondered if the environment in which tardigrades live may be correlated to their thermal tolerance. Qualitatively there might appear to be some correlation between the site of original collection or culture conditions and a tardigrades ability to survive heating. For example, the *A. antarcticus*, which was originally collected in the cold and dry Antarctica, and cultured at 5°C, performed worse, especially in the dry state, when exposed to heat stress than *V. viridianus*, which was collected from a warm and humid site in Alabama, USA. However, a correlative analysis comparing temperature and precipitation levels of the collection site for each species and the species' LT50 values indicates no significant relationship between these parameters (Fig. 5A-F). To take into account the fact that cultured species may not be living in the same conditions as their environmental collection, we also used culture temperatures (for cultured species) to look for correlations in survival. We found that these comparisons also yielded no significant correlations.

## DISCUSSION

Thermal stress exerts myriad perturbations to organisms, cells, and biological macromolecules that have evolved to function within a typically confined range of temperatures. Increased temperatures can disrupt or perturb membrane fluidity, protein folding and interactions, metabolic rates, diffusion, and a host of other biological and physical processes that are essential for life (Altschuler and Mascarenhas, 1982; Kühl and Rensing, 2000). As global temperatures rise, it becomes ever more pressing to understand not only the effects that increased temperatures exert on organisms, but also how extremo-tolerant organisms, such as tardigrades, might cope with such changes.

Here we confirm that contrary to their reputation as one of the ‘toughest’ animals, tardigrades in an active state are relatively susceptible to even mild thermal stress. In their active state, tardigrades can only tolerate these mild increases in temperature for a short duration and exposure to these mild thermal fluctuations does not precondition them to survive subsequent heating at higher temperatures. Interestingly, thermal tolerance was not uniform among the six species of tardigrades tested here, with differences in max temperature and lethal temperature 50 observed in both the active and dry state across species.

Consistent with past observations (Rebecchi et al., 2009b), we found that in several cases response to stimuli and coordinated movement increased at 72 h versus 1 h post-heating. To date, no systematic studies have been performed to assess metabolism in heat shocked tardigrades, and thus it is unknown whether this apparent lifelessness is true cryptobiosis (suspension of all life processes). Previous reports have suggested that during heating tardigrades may experience anoxia and/or hypoxia due to lower oxygen levels in their culture media (Rebecchi et al., 2009b). If true, anoxybiosis may account for the temporary suspension of processes such as movement and response to stimuli. It is of note that under low oxygen conditions tardigrades become turgid with their appendages outstretched (Guidetti et al., 2011a,b) as was observed in each of the species tested here. However, here 1 ml of fresh water was added to the tardigrades post-heating, which would allow for at least partial reoxygenation, which one might reasonably expect to allow for tardigrades to come out of turgor. Further studies will need to be done in order to assess whether anoxia is directly linked to the suspension of movement and response to stimuli upon heating in tardigrades.

Attempted preconditioning, under the conditions used here, did not improve tardigrade thermal tolerance. It is possible that under different conditions than the ones used here preconditioning might improve thermal tolerance. The conditions used here were as follows: tardigrades were treated with a mild stress for 45 min, followed by a recovery time of 1 h, and finally exposure to the more severe thermal stress for 45 min. Forty-five minutes was chosen as an exposure time for the mild stress since induction of an average gene's transcription takes 5-15 min (Tullai et al., 2007), the average time it takes to begin translating an mRNA is typically 2-5 min (Schwanhäusser et al., 2013), and the average time for induction of stress-related metabolites is ∼30 min (Hottiger et al., 1987). Similarly, while we only tested preconditioning with a 1-h recovery period, we believe that this period is appropriate when one considers that the average half-life for a metabolite is often reported as ∼1-2 h (Park et al., 2016), ∼9 h for a typical mRNA (Friedel and Dölken, 2009), ∼46 h for a typical protein (Schwanhäusser et al., 2013). This means pre-exposure for 45 min and a recovery time of 1 h should allow for the enrichment of most responsive molecules, while not allowing them sufficient time to be turned over. Of course, these are average induction and turnover times and without knowing the exact mediators of tardigrade thermal tolerance it is not possible to say for sure if these times are appropriate. Further research into the mediators of thermal tolerance will shed light on their ability to precondition tardigrades to survive high temperatures.

There appears to be no correlation between tardigrade class and thermal tolerance in either the hydrated or dry state, as eutardigrade species displayed both the lowest (*A. antarcticus* at 1 h and *Milnesium* sp*.* at 72 h) and the highest (*H. exemplaris*, *R. varieornatus, V. viridianus*) thermal tolerances. The heterotardigade, *Echiniscus* sp*.*, also survived heating in both the dry and hydrated state to a middling degree. Similarly, the lifestyle (limnic/freshwater versus limno-terrestrial) of a tardigrade species did not appear to be a determining factor in thermal tolerance as the fresh-water aquatic species, *H. exemplaris*, survive as well as many of the limno-terrestrial species (e.g. *Echiniscus* sp*.,* or *Milnesium* sp*.*) in both the hydrated and dry state. Our study did not include marine species, and further investigation into the thermal tolerance of tardigrades inhabiting these environments should be performed. Furthermore, limitations in the correlative analysis between environmental factors at the site of collection and tolerated temperatures exist. For example, *H. exemplaris* is derived from a fresh water pond whose daily fluctuations in temperature would be less than the surrounding environment. Alternatively, *R. varieornatus* was collected from moss growing on pavement, an environment that would be expected to heat up much more than the surrounding environment. Though beyond the scope of this current study, future investigation of microenvironmental fluctuations and tardigrade tolerances will be a valuable addition to the knowledgebase of the field.

In our study *A. antarcticus* was reared at 5°C, that is a different condition with respect to the reared temperature of 15°C used in a prior study that examined thermal tolerance in the same species (Giovannini et al., 2018). This temperature was originally selected to test whether culture temperature had an effect on maximal temperature tolerance, however, we found little difference between specimens reared here (5°C) and those previously assayed (15°C) (Giovannini et al., 2018). In the hydrated state, *A. antarcticus* specimens reared at 5°C and 15°C tolerated heating to a similar maximal temperature of 34-35°C measured after 1 h of recovery, or 37-38°C as measured after 24-72 h of recovery. This is of note as Li and Wang (2005a) found that acclimatizing of the tardigrade, *Macrobiotus harmsworthi*, to different temperatures results in a change in preferred temperature, but no change in maximal tolerated temperature (Li and Wang, 2005a). Our study supports and expands to other species the notion that while preferred temperature may change with acclimatization, maximal tolerated temperature does not.

On a molecular level the mediators involved in tardigrade thermal tolerance are not well understood. Further research will be required to understand whether mediators or mechanisms of other stress tolerances, such as heat shock proteins (Al-Ansari et al., 2024; Hibshman et al., 2023; Neves et al., 2022), modulated lipid metabolism (Ren et al., 2020), tardigrade disordered proteins (Biswas et al., 2024; Boothby et al., 2017; Hesgrove and Boothby, 2020; Nguyen et al., 2022; Piszkiewicz et al., 2019; Sanchez-Martinez et al., 2023, 2024; Tanaka et al., 2015; Yamaguchi et al., 2012), reactive oxygen species scavengers (Giovannini et al., 2022) or late embryogenesis abundant proteins (Kc et al., 2024 preprint; Li et al., 2024; Tanaka et al., 2015) might help confer tolerance to thermal stress in tardigrades.

Since tardigrades' tolerance to increased temperature is modest at best under hydrated conditions and improves dramatically in most species when dry, there appears to be a mechanistic link between drying and high thermal tolerance. Future studies will need to be conducted to directly confirm this hypothesis. Previous studies have suggested that glass transition temperature may limit thermal tolerance of tardigrades (and other organisms) in the dry state (Boothby, 2019, 2021; Boothby et al., 2017; Hengherr et al., 2009; Sakurai et al., 2008; Sun and Leopold, 1997), though this notion is not fully accepted or refuted for tardigrades (Arakawa and Numata, 2021; Boothby, 2021). In cases where glass formation, or vitrification, is employed, it will be important to look at other properties beyond glass transition temperature, as recent studies suggest that additional properties of glassy systems correlate with protective capacity under stress conditions (Kumara et al., 2024; Ramirez et al., 2023).

We would note that when we describe the tardigrades' ability to survive heating as ‘mild’ this is an anthropomorphic view. Even though the tardigrades used in this study clearly do not display extremophilic or extremotolerant characteristics with regard to heating in the active state, for small aquatic animals heating to 35-40°C likely represents the extreme high end of temperatures they would encounter naturally in an active state. Particularly, Antarctic species may never naturally experience such temperatures.

Overall, our study provides new insights into the extent of thermal tolerance in active and anhydrobiotic tardigrades. We find that in their hydrated state tardigrades survive over a relatively small range of temperatures and that even with preconditioning or repeated exposure they survive the same temperatures. For this reason, tardigrades have the potential to be a good bioindicator species. Bioindicator species are species that are used to assess the quality of the environment and how it changes over time. Good bioindicators display fitness across a moderate environmental gradient, in this case temperature (Holt and Miller, 2011). Insight into tardigrade's temperature preference can help us to further understand the temperature impacts of climate change on the environment where these species live. Additionally, the observation that in a dry state thermal tolerance is enhanced in many species of tardigrades provides potential insights into how thermal tolerance might be engineered in the future (e.g. to generate heat tolerant crops) through the induction of transient biostasis.

## MATERIALS AND METHODS

### Specimen collection and identification

Six species of tardigrades were selected for the experiments: *A. antarcticus*, *H. exemplaris*, *R. varieornatus*, *Milnesium* sp*.*, *Echiniscus* sp*.*, and *V. viridianus*. Three species *A. antarcticus* (Cesari et al., 2016), *H. exemplaris* (Gabriel et al., 2007), and *R. varieornatus “*YOKOZUNA-1*”* (Horikawa et al., 2008) were extracted from cultures (see Table 2 for collection date and site), and three species (*Milnesium* sp*.*, *Echiniscus* sp*.*, and *V. viridianus*) were identified and extracted from environmental lichen samples throughout the U.S. (see Table 2 for geographical data).

**Table 2.**
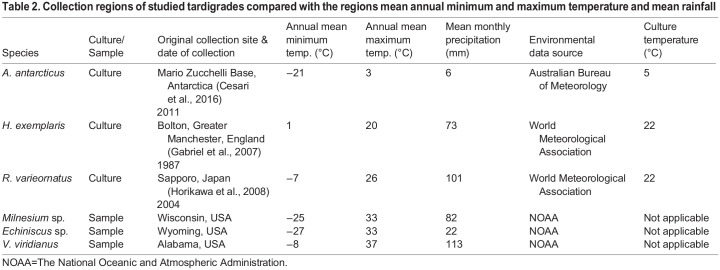
Collection regions of studied tardigrades compared with the regions mean annual minimum and maximum temperature and mean rainfall

*H. exemplaris* and *R. varieornatus* were cultured in bottled spring water (Deer Park) directly supplemented with super-fresh chlorella V12 (Reed Mariculture) at room temperature (21°C) as described in Horikawa et al. (2008). *A. antarcticus* was cultured as previously described in Giovannini et al. (2018) with a slight deviation in that specimens in our study were reared at 5°C, not 15°C as previously used in Giovannini et al. (2018).

Tardigrades collected from environmental samples were identified through a combined approach utilizing phase contrast compound microscopy (Nikon Eclipse E600), dichotomous keys, morphometrics, and world literature for morphological analysis (Degma and Guidetti, 2024; Gąsiorek et al., 2019; Morek et al., 2019; Ramazzotti and Maucci, 1995). DNA barcoding and BLAST were utilized for genetic analysis of tardigrades extracted from environmental samples (Altschul et al., 1990).

### Specimen identification and BLAST results

The specimens of *V. viridianus* used in the current study were extracted, identified, and cultured from the same lichen sample used in a previous study (Momeni et al., 2022, 2023). DNA barcoding of the 28S rRNA gene was used to identify the *Echiniscus* sp. and *Milnesium* sp. extracted from lichen samples for this study (Dabert et al., 2014; Guil et al., 2015). Sequences of the 28S rRNA gene can be found in file S1. All DNA extractions and PCR amplifications were performed as described in (Stec et al., 2015) with the one modification that up to ten tardigrades were used for DNA extractions rather than a single animal.

### Active heat tolerance assays

Three replicates of ten active tardigrades of each species were tested at each temperature. For heating, a heat block was set to the following temperatures: 55°C, 60°C, 65°C, 70°C, 75°C, 80°C, 85°C, and 90°C. For controls, specimens were kept at 21°C. Each replicate of ten tardigrades was placed in a 35×10 mm Petri dish on 3 ml of solidified 2% agar with 3 ml of Arrowhead Natural Spring Water. After that, the Petri dishes were placed on an IsoBlock Digital dry heater (Benchmark Scientific BSH6000) set to the corresponding trial temperature and heated for 45 min. Once heated for 45 min, the temperature of water in the Petri dishes was measured empirically with a digital thermometer with final temperatures reached being 34°C, 35°C, 36°C, 38°C, 41°C, 43°C, 45°C, and 47°C. Petri dishes were removed from the heater and 1 ml of the heated water was replaced with 1 ml of fresh, room temperature spring water. Observations for tardigrade activity and response to stimuli were documented 1 h and 72 h after exposure. These times were selected to provide a short-term and long-term observation point. 72 h was used as a long-term time point rather than 24 or 48 h to ensure that animals marked as surviving after 1 h are truly capable of long-term survival. To gauge response to stimuli, animals were placed in clear dishes above a light source for 1 h prior to observation.

Additionally, the most heat-resistant species (*V. viridianus*) was later selected for heating trials for varying time periods at 38°C since this was the highest temperature where over half of the individuals survived. Time periods for heating included 45 min, 1 h, 6 h, and 24 h. Three replicates of ten active *V. viridianus* were placed in Petri dishes on 2% agar with 3 ml of natural spring water, then placed on a heating block set to 70°C (per previous measurements) for each of the selected time periods. Spring water in a 250 ml flask placed on the heating block set to 70°C was aliquoted to the Petri dishes periodically to ensure the water did not evaporate and tardigrades were completely submerged for the entire period. For testing the 24 h time period, three replicates of ten active *V. viridianus* were placed in a 250 ml flask with 2% agar and 200 ml of natural spring water. After each time period, Petri dishes were removed and 1 ml of heated water was replaced with 1 ml of fresh, room temperature spring water. For the 24 h period, tardigrades were washed out of the flask, then separated into three Petri dishes with 3 ml of fresh, room temperature natural spring water. Observations for tardigrade activity were made at 1 h and 72 h after being given fresh water and activity (coordinated movements with or without response to stimuli performed with light) count was recorded.

*V. viridianus* was also selected to be tested with repeated heat shocks at 38°C, 41°C, and 43°C. We continued using three replicates of ten active *V. viridianus* for each measurement. We tested three repetitive heat treatments with either 1 h of recovery between treatments or 72 h of recovery between treatments. Tardigrades were separated into Petri dishes, each dish containing 3 ml of spring water and ten active tardigrades. These dishes were then places on a heat block set to the desired temperature for 45 min. For all tardigrades, they were given 1 ml of fresh water after being heated and then left at room temperature to recover. Tardigrades that were given 1 h to recover were then heated twice more following the same recovery procedure between heating. Their activity was recorded 72 h after their final round of heating. Tardigrades given 72 h to recover were left at room temperature between heating, otherwise they received the same treatment as the 1 h recovery group. Given that allowing for 72 h of recovery between treatments meant that tardigrades were left on the bench without food for 9 days, the data from this group were normalized to a control group of unheated tardigrades left at room temperature without algae for the same period of time.

Furthermore, we selected *V. viridianus* to be tested for the possibility of being conditioned to survive higher temperatures than originally observed. We chose the temperatures 35°C, 36°C, 38°C, and 41°C as conditioning temperatures to then see if they could survive a 47°C treatment, at which they all died in the original observation. We continued using three replicates of ten active *V. viridianus* for each measurement. We heated them at either 35°C, 36°C, 38°C, or 41°C first for 45 min, then replaced 1 ml of the water with fresh natural spring water, provided photostimulation and an incubation period of 1 h, checked and recorded activity, then heated them again at 47°C for 45 min, replaced the water, and checked and recorded activity after 1 h incubation period and photostimulation. Another 1 ml of fresh water and 1 h of photostimulation were provided before checking the activity count again at the 72 h mark.

### Desiccated heat tolerance assay

We used the same six species of tardigrade to test tolerance to heat in a desiccated state. Temperatures chosen for this test were 55°C, 65°C, 75°C, 85°C, 95°C, 105°C, 115°C, and 125°C. For each taxon and temperature treatment, three replicates of ten individuals were selected. Whatman filter paper was cut into small squares, then taped with double sided Scotch tape to the inside bottom of the 35×10 mm Petri dishes. Individuals were transferred from their respective samples or cultures to Whatman filter paper using a p20 pipette. Specimens were left to desiccate on the filter paper at room temperature/humidity (21°C/34% RH) for 1 h, then specimens were entered into a desiccation chamber at room temperature with drierite (W.A. Hammond Drierite Co.21005) at 5% RH for 1-24 h. After that, the desiccated specimens in Petri dishes were entered into an Espec BTL-433 Temperature and Humidity Chamber set to the corresponding treatment temperatures at 10% humidity and left inside for 45 min. After 45 min, specimens were removed from the Espec and hydrated with 3 ml of natural spring water. Activity was recorded after 1 h of hydration and photostimulation. The specimens were provided an extra 1 ml of natural spring water and provided 1 h of photostimulation right before checking their activity at the 72-h mark, as in the previous assays. For controls, specimens were desiccated and rehydrated at 21°C to check their activity after 1 h and 72 h after rehydration.

### Controls

All control (room temperature) treatments were performed concurrently with experimental temperature treatments for both hydrated and desiccated animals. The one exception to this were control treatments of dry *A. antarcticus* specimens. In this case, dry *A. antarcticus* specimens were tested for survival at room temperature and after observing 100% survival in all three biological replicates we moved on to testing this species under different temperatures.

### Statistical analyses

For all experiments three replicates of ten individuals were used. The mean survival across three replicates is reported with error bars showing standard deviation. The scripting software R and packages were used for conducting statistical tests and creating graphs for data visualization. Statistical tests conducted to analyze data include one-way ANOVAs with Tukey's post hoc tests. Correlations shown in Fig. 5 were made using Spearman's rank correlation test.

## Supplementary Material

10.1242/biolopen.060485_sup1 Supplementary information

File S1. rRNA sequences generated in this study.

File S2. Data used and analyzed in this study.

File S3. Scripts and code used in this study.
