# Supplementary material for: An evaluation of thermal tolerance in six tardigrade species in an active and dry state
Source: Biol Open. 2024 Sep 25;13(10):bio060485. doi: 10.1242/bio.060485 (PMC11451804; doi:10.1242/bio.060485)
Supplement: Supplementary information [file biolopen-13-060485-s1.pdf]

File S1. rRNA sequences generated in this study.

Available for download at
https://journals.biologists.com/bio/article-lookup/doi/10.1242/bio.060485#supplementary-data

File S2. Data used and analyzed in this study.

Available for download at
https://journals.biologists.com/bio/article-lookup/doi/10.1242/bio.060485#supplementary-data

File S3. Scripts and code used in this study.

Available for download at
https://journals.biologists.com/bio/article-lookup/doi/10.1242/bio.060485#supplementary-data

Biology Open • Supplementary information
